# Long-term alterations in gut microbiota following mild COVID-19 recovery: bacterial and fungal community shifts

**DOI:** 10.3389/fcimb.2025.1565887

**Published:** 2025-05-26

**Authors:** Da Li, Da-Ya Zhang, Shi-Ju Chen, Yan-Ting Lv, Shi-Mei Huang, Chen Chen, Fan Zeng, Run-Xiang Chen, Xiao-Dong Zhang, Jian-Xin Xiong, Fa-Di Chen, Yue-Hong Jiang, Zhai Chen, Cui-Yi Mo, Jia-Jia Chen, Xu-Li Zhu, Li-Jun Zhang, Fei-Hu Bai

**Affiliations:** ^1^ The Second School of Clinical Medicine, Hainan Medical University, Haikou, China; ^2^ Department of Gastroenterology, Hainan Second People’s Hospital, Wuzhishan, China; ^3^ Department of Gastroenterology, Wuzhishan Center for Disease Control and Prevention, Wuzhishan, China; ^4^ Department of Gastroenterology, The Second People ‘s Hospital of Ledong Li Autonomous County, Ledong Li Autonomous County, China; ^5^ Department of Gastroenterology, Dongfang People’s Hospital, Dongfang, China; ^6^ Department of Gastroenterology, Qionghai People’s Hospital, Qionghai, China; ^7^ Departmenrt of Internal Medicine, Otog Front Banner People ‘s Hospital, Otog Front Banner, China; ^8^ Health Management Center, The Second Affiliated Hospital of Hainan Medical University, Haikou, China; ^9^ Department of Gastroenterology, The Second Affiliated Hospital of Hainan Medical University, Haikou, China; ^10^ Department of Gastroenterology, The Gastroenterology Clinical Medical Center of Hainan Province, Haikou, China

**Keywords:** mild COVID-19, gut microbiome, metagenomic sequencing, probiotics, fungal microbiota, random forest model, ROC curve analysis, bacterial-fungal co-occurrence network

## Abstract

**Objective:**

COVID-19 has had a profound impact on public health globally. However, most studies have focused on patients with long COVID or those in the acute phase of infection, with limited research on the health of individuals who have recovered from mild COVID-19. This study investigates the long-term changes in bacterial and fungal communities in individuals recovering from mild COVID-19 and their clinical relevance.

**Methods:**

Healthy individuals from Hainan Province were enrolled before the COVID-19 outbreak, along with individuals recovering from COVID-19 at 3 months and 6 months post-recovery. Stool, blood samples, and metadata were collected. Metagenomic sequencing and Internal Transcribed Spacer (ITS) analysis characterized bacterial and fungal communities, while bacterial-fungal co-occurrence networks were constructed. A random forest model evaluated the predictive capacity of key taxa.

**Results:**

The gut microbiota of COVID-19 recoverees differed significantly from that of healthy individuals. At 3 months post-recovery, probiotics (e.g., *Blautia massiliensis* and *Kluyveromyces* spp.) were enriched, linked to improved metabolism, while at 6 months, partial recovery of probiotics (e.g., *Acidaminococcus massiliensis* and *Asterotremella* spp.) was observed alongside persistent pathogens (e.g., *Streptococcus equinus* and *Gibberella* spp.). Dynamic changes were observed, with *Acidaminococcus massiliensis* enriched at both baseline and 6 months but absent at 3 months. Co-occurrence network analysis revealed synergies between bacterial (*Rothia* spp.) and fungal (*Coprinopsis* spp.) taxa, suggesting their potential roles in gut restoration. The bacterial random forest model (10 taxa) outperformed the fungal model (8 taxa) in predicting recovery status (AUC = 0.99 vs. 0.80).

**Conclusion:**

These findings highlight the significant long-term impacts of mild COVID-19 recovery on gut microbiota, with key taxa influencing metabolism and immune regulation, supporting microbiome-based strategies for recovery management.

## 1 Introduction

Since the initial outbreak of SARS-CoV-2 in December 2019, COVID-19 has emerged as a significant global public health challenge, resulting in millions of infections and deaths worldwide (Collaborators GUBoD, 2024; Quinlan et al., 2024; Zhang et al., 2024). Although mild COVID-19 cases typically do not result in severe acute outcomes, increasing attention is given to their potential long-term health effects (Hacker et al., 2021; Fitzgerald et al., 2024). Existing studies suggest that even mild COVID-19 can lead to prolonged multisystem impacts, including potential effects on the gastrointestinal tract (Mizrahi et al., 2023; Kim et al., 2024; Porter et al., 2024; Taquet et al., 2024; Trender et al., 2024; Zhang et al., 2024). Increasing evidence indicates that SARS-CoV-2 can directly infect and replicate within the gut, with the interaction between the virus and ACE2 receptors on intestinal epithelial cells identified as a key mechanism (Pola et al., 2021; Zhang et al., 2022; Smyth et al., 2023; An et al., 2024). Viral RNA has been detected in stool samples from approximately 50% of patients, and gastrointestinal symptoms, such as diarrhea and nausea, have been reported even in mild cases (Liang et al., 2020; Daou et al., 2022; Zhou et al., 2022).

**Figure 1:**
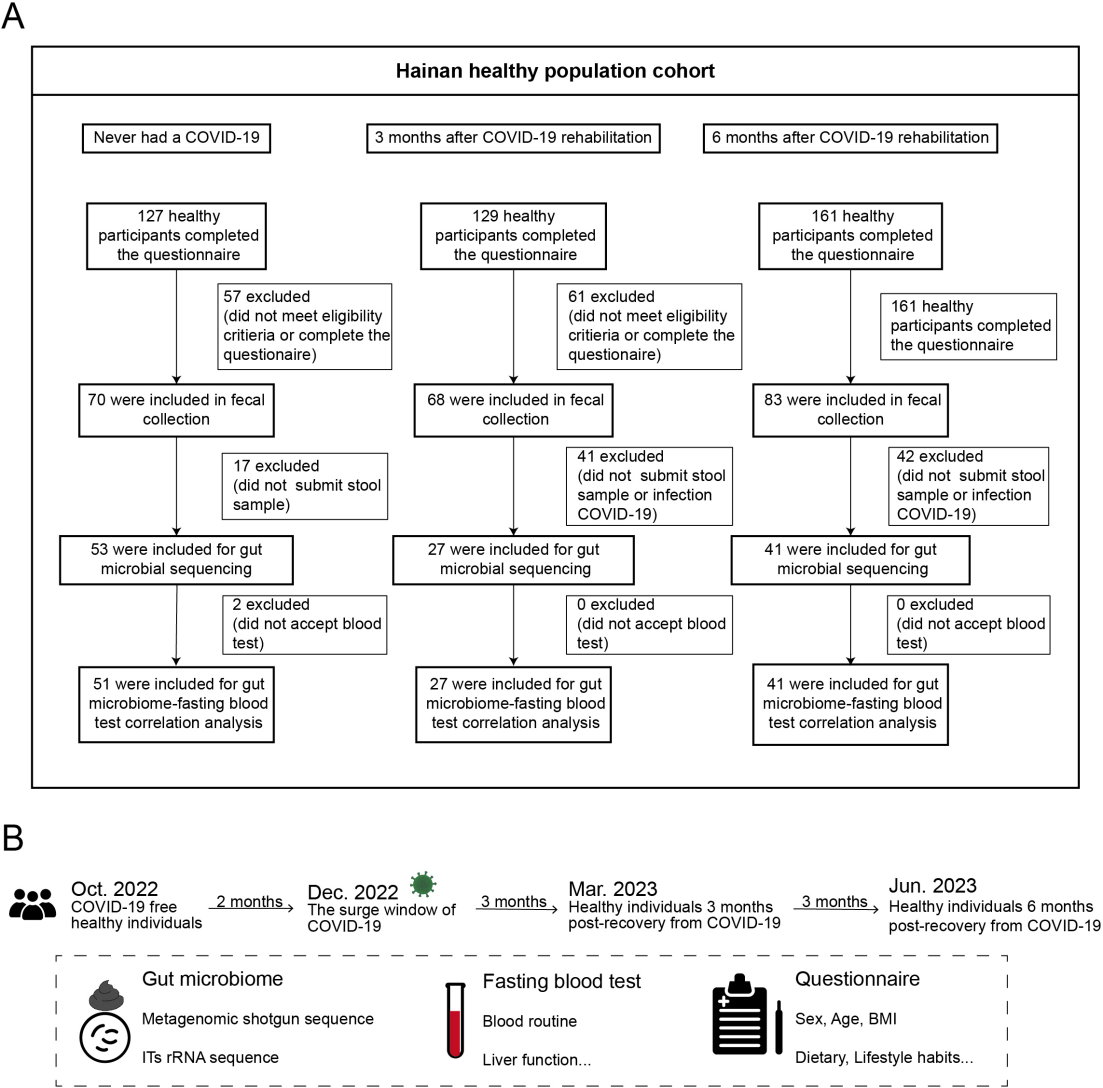
Study design and cohort recruitment timeline for COVID-19 recovery analysis. **(A)** Three healthy cohorts were recruited: 53 individuals who never had COVID-19, 27 individuals 3 months post-recovery, and 41 individuals 6 months post-recovery. **(B)** Timeline of recruitment, sample collection, and follow-up before COVID-19 and at 3 and 6 months post-recovery.

While emerging longitudinal studies have begun to elucidate the dynamics of post-COVID gut microbiota (Chen et al., 2022; Upadhyay et al., 2023), significant knowledge gaps remain concerning the microbial sequelae following mild infections. Chen et al. (Gut, 2022) reported partial restoration of bacterial α-diversity indices at 6 months post-infection in moderate cases (Shannon index Δ=0.38, P=0.017) (Chen et al., 2022), whereas Upadhyay et al. (mBio, 2023) identified persistent β-diversity distortions in mild cases during a 9-month surveillance period (Bray-Curtis R²=0.12, P<0.001) (Upadhyay et al., 2023). Our systematic characterization across key convalescent windows (3 and 6 months) reveals non-linear recovery trajectories (**Figure 1B**), offering a broader perspective than the cross-sectional analysis conducted by Su et al. (Cell Host Microbe, 2024) (Su et al., 2024). Three critical limitations in the current understanding of post-COVID microbiota dynamics warrant particular attention: First, despite increasing recognition of fungal contributions to mucosal immunity (Bandala-Sanchez et al., 2022; Liao et al., 2024a), prior analyses have predominantly focused on bacterial communities (Yeoh et al., 2021; Chen et al., 2022; Upadhyay et al., 2023), a limitation addressed by our identification of time-specific mycobiome signatures. Second, the geographical generalizability of microbial signatures remains unestablished (Kopera et al., 2024), a gap mitigated in our study through a dual-cohort framework that compares tropical and temperate populations. Third, while correlations between microbial and blood parameters have been hypothesized (Su et al., 2024), their clinical translatability remains unvalidated. This gap is bridged here through robust metabolic associations and high-accuracy predictive modeling.

The human gut microbiota, composed of bacteria, fungi, and viruses, plays a vital role in host health, particularly in immune regulation, nutrient metabolism, and pathogen defense (Lo et al., 2021; Afzaal et al., 2022; Ezzatpour et al., 2023; Huang et al., 2024; Ignacio et al., 2024; Thapa et al., 2024). Dysbiosis, an imbalance in the gut microbiota, has been associated with various chronic diseases, including inflammatory bowel disease, metabolic syndrome, and neurological disorders (Gao et al., 2021; Ezzatpour et al., 2023; Buttar et al., 2024; de Jonge et al., 2024; Malan-Müller et al., 2025). In recent years, the significance of the gut fungal community has gained increasing recognition, particularly in immune modulation and maintaining gut barrier integrity (Bandala-Sanchez et al., 2022; Khalil et al., 2024; Liao et al., 2024b; Li et al., 2024). The complex interactions between bacteria and fungi can synergistically support host homeostasis, but when disrupted, they may contribute to inflammation and metabolic disturbances (Intarajak et al., 2024; Zhang et al., 2024).

Previous studies have reported significant alterations in the gut microbiota of COVID-19 patients following infection, including reduced bacterial diversity, decreased probiotics, and increased pathogenic bacteria (Yeoh et al., 2021; Chen et al., 2022; Upadhyay et al., 2023; Kopera et al., 2024; Li et al., 2024; Su et al., 2024). Additionally, changes in the gut fungal community may further influence host immune responses and modulate inflammation during recovery (Flemming, 2023; Rizzello et al., 2024). However, research on long-term changes in gut microbiota after recovery from mild COVID-19, particularly regarding bacterial and fungal diversity and their functional relevance, remains limited. Furthermore, the relationship between post-recovery microbial changes and delayed symptoms associated with long COVID syndrome has not been systematically analyzed.

To address these gaps, this study compares the gut microbiota composition and diversity in healthy individuals who never contracted COVID-19 with those recovering from mild COVID-19 at 3 months (C3M) and 6 months (C6M) post-recovery. The study aims to identify key microbial features and evaluate their potential in predicting post-recovery health outcomes by analyzing dynamic changes in bacterial and fungal communities and their correlations with clinical indicators. These findings may offer new insights into post-COVID-19 health management and microbiota-targeted therapeutic strategies.

## 2 Methods

### 2.1 Cohort description, subject selection, and sample collection

In December 2022, before the peak of COVID-19 prevalence in China, a gut microbiota survey was conducted among 53 healthy individuals in Hainan Province. These participants underwent daily COVID-19 nucleic acid testing, all yielding consistently negative results, and had no prior history of COVID-19 infection. Between February and June 2023, another gut microbiota survey was conducted among the general population in Hainan, enrolling 27 participants who had recovered from COVID-19 for 3 months and 41 participants who had recovered for 6 months (**Figure 1A**). These recovered participants had positive SARS-CoV-2 nucleic acid results from throat swabs, exhibited typical symptoms such as cough and fever during the acute phase, and were clinically classified as mild or ordinary cases according to the Chinese “Guidelines for Diagnosis and Treatment of COVID-19 Infection (Trial Version 10).” All participants had received two doses of the COVID-19 vaccine prior to the study. None of the participants showed symptoms of long COVID, such as fatigue or headache, after recovery. Fecal and blood samples were collected from all participants. Abdominal ultrasounds were performed, and detailed metadata were gathered through questionnaires, including baseline information such as height, weight, gender, age, and chronic disease status. Lifestyle factors (e.g., physical activity, daily routines, and sleep duration) and dietary habits (e.g., food preferences, appetite, and body weight) remained consistent for all participants over one year of follow-up. None of the participants had received antibiotic treatment in the past three months. All recovered participants had experienced only one infection during the COVID-19 outbreak period in China (**Figure 1B**). Fecal samples were preserved in dry ice within 30 minutes and stored at −80°C within 24 hours, while blood samples were stored at 4°C and analyzed within 6 hours. Ethical approval was obtained from the Medical Ethics Committee (LW2022270), and informed consent was signed by all participants. This study was conducted in accordance with the ethical standards of the Medical Ethics Committee and the 1964 Helsinki Declaration and its later amendments.

### 2.2 Fasting blood tests

Venous blood samples were collected from all participants in the morning after an overnight fast. Blood tests included routine blood tests, liver function, electrolytes, glucose, and lipid profiles. Routine blood parameters, including red blood cell count (RBC), white blood cell count (WBC), hemoglobin (HGB), hematocrit (HCT), mean corpuscular volume (MCV), and platelet count (PLT), were measured using an automated hematology analyzer (e.g., Sysmex XN-9000, Japan). Liver function parameters, including alanine aminotransferase (ALT), aspartate aminotransferase (AST), alkaline phosphatase (ALP), total protein (TP), albumin (ALB), total bilirubin (TBIL), and direct bilirubin (DBIL), were analyzed using an automated biochemical analyzer (e.g., Roche Cobas 8000, Switzerland). Electrolytes such as sodium (Na^+^), potassium (K^+^), chloride (Cl^-^), calcium (Ca²^+^), and phosphate (P) were measured using an electrolyte analyzer (e.g., Beckman Coulter AU680, USA). Fasting plasma glucose (FPG) and lipid profiles, including total cholesterol (TC), triglycerides (TG), high-density lipoprotein cholesterol (HDL-C), and low-density lipoprotein cholesterol (LDL-C), were also measured using the same biochemical analyzer. All procedures followed the manufacturer’s instructions, and results were interpreted by trained professionals for subsequent analyses.

### 2.3 Fecal microbiome DNA extraction and metagenomic sequencing

Fecal microbial DNA was extracted using the TIANamp Stool DNA Kit (#DP328) according to the manufacturer’s protocol. Briefly, approximately 100 mg of stool sediment was added to a ZR BashingBead™ lysis tube containing 750 μL of lysis buffer and processed at maximum speed for 10 minutes. The lysate was then centrifuged at ≥10,000g for 1 minute, and the supernatant was transferred to a Zymo™ Spin™ III-F filter in a collection tube, followed by centrifugation at 8,000g for 1 minute. Three volumes of genomic lysis buffer were added to the filtrate, which was purified in a new spin column. Eluted DNA (50 μL; 20–300 ng/μL) was used for metagenomic sequencing, which was performed by SHANGHAI BIOTREE BIOTECH CO., LTD.

### 2.4 Fecal microbiome metagenomic shotgun sequencing and data processing

Sequencing libraries were prepared using the Illumina^®^ Rapid Plus DNA Library Prep Kit following the manufacturer’s protocol. Library quality was assessed using a Qubit^®^ 3.0 fluorometer (Invitrogen, USA). Sequencing was conducted on the Illumina platform in PE150 mode (Magigene Biotech), generating 151 bp paired-end reads with an average of 62.1 ± 8.4 million reads per sample. Raw reads were processed using Trimmomatic (v.0.39) to remove low-quality sequences and generate clean data for downstream analysis. Taxonomic profiling of metagenomic samples was performed using MetaPhlAn2, which quantifies taxa at the species level using clade-specific markers. MetaPhlAn2 was run with default parameters and the marker gene database CHOCOPhlAnSGB 202103).

### 2.5 Fecal microbiome data analysis

Microbiome abundance tables were converted to relative abundance for downstream analysis. Beta diversity differences between groups were assessed using principal coordinate analysis (PCoA) based on Bray-Curtis dissimilarity. PERMANOVA tests were conducted to determine the statistical significance of microbiome composition differences between groups at various time points. Alpha diversity was assessed using the Shannon index to compare microbiome diversity across groups. Differentially abundant species were identified using linear discriminant analysis effect size (LEfSe) and were further analyzed for phylogenetic relationships. The gut microbiota of healthy individuals before COVID-19 (NC) served as the control group and was compared with the post-COVID-19 recovery groups to evaluate the long-term effects of the disease on gut microbiota.

### 2.6 ITS DNA extraction and sequencing

Total fecal microbial DNA was extracted using a Magnetic Soil and Stool DNA Kit (DP712-02, TIANGEN, China) following the manufacturer’s instructions. DNA quantification was performed with a Qubit fluorometer (Invitrogen, USA). PCR amplification was performed using universal primers ITS1FI2/ITS2 (ITS1FI2: 5′-GTGARTCATCGAATCTTTG-3′; ITS2: 5′-TCCTCCGCTTATTGATATGC-3′). Amplification conditions included an initial denaturation at 95°C for 30 seconds, followed by 32 cycles of denaturation at 95°C for 10 seconds, annealing at 52°C for 30 seconds, and extension at 72°C for 45 seconds, with a final extension at 72°C for 10 minutes. PCR products were purified using AMPure XT Beads (Beckman Coulter Genomics, Danvers, MA, USA) and quantified using Qubit. Qualified products were assessed using an Agilent 2100 bioanalyzer (Agilent, USA) and sequenced on an Illumina NovaSeq 6000 (PE250) by SHANGHAI BIOTREE BIOTECH CO., LTD.

### 2.7 ITS sequencing data processing and analysis

Sequencing primers were trimmed from raw demultiplexed sequences using cutadapt (v1.9). Paired-end reads were merged using FLASH (v1.2.8). Low-quality reads (quality score <20), short reads (<100 bp), and reads with >5% “N” were filtered using a sliding window algorithm (fqtrim v0.94). Chimeric sequences were removed using Vsearch (v2.3.4). Denoising and amplicon sequence variant (ASV) generation were performed using DADA2. Taxonomic annotation was conducted with QIIME2’s feature-classifier plugin against the RDP and UNITE databases. Linear discriminant analysis effect size (LEfSe; LDA≥3.0, P<0.05) was performed using nsegata-lefse, and other visualizations were created using R packages (v3.4.4).

### 2.8 Random forest and ROC curve analysis

Random forest analysis was performed using R software to analyze differential bacterial (10 species) and fungal (8 genus) taxa between healthy individuals without COVID-19 and those who had recovered from COVID-19 for 6 months in the Hainan cohort. Receiver operating characteristic (ROC) curves were plotted to evaluate the predictive capacity of these taxa for COVID-19 recovery status. To validate the findings, a separate cohort from Ordos, Inner Mongolia, was recruited between April and June 2023, following the same inclusion criteria and sample collection methods as the Hainan cohort. The Inner Mongolia cohort comprised 153 participants, including 42 healthy individuals and 111 post-COVID-19 recovery participants. The model was trained on the Hainan cohort and validated independently on the Inner Mongolia cohort. Further validation was performed using combined data from both cohorts. Prior to model fitting, the threshold for exclusion was set to 1% relative abundance across all samples, and continuous data from both macrogenomic and ITS datasets were Z-Score normalized. Random forest modeling was performed using the R package “randomForest”, with feature selection conducted via the rfcv function (10-fold cross-validation, repeated 5 times). The top 20 features were selected based on MeanDecreaseAccuracy. Data were randomly stratified into 7:3 training and testing sets, and the model was trained with treeNum set to 5000. ROC curve analysis using the pROC package evaluated the model’s predictive capacity for COVID-19 recovery status. The methodology was applied to the Hainan cohort (training) and the Inner Mongolia cohort (independent validation), with combined cohort data used for further validation.

## 3 Results

### 3.1 Cohort description

In the coastal regions of southern China, particularly in Hainan Province, limited data were available on the interaction between SARS-CoV-2 infection and the gut microbiome. To fill this gap, a cross-sectional study was conducted to collect fecal samples from 121 healthy individuals. The cohort consisted of three groups: 53 participants who had never contracted COVID-19 prior to the outbreak (NC group), 27 participants who were three months post-COVID-19 recovery (C3M group), and 41 participants who were six months post-recovery (C6M group) (**Figure 1B**). The three groups were similar in age, with mean ages ranging from 41 to 46 years, and had comparable body mass index (BMI), ranging from 22.9 to 24.2 (**Table 1**). The C6M group had a slightly higher proportion of females (33 participants, 80.2%), while the NC and C3M groups showed more balanced gender distributions. PERMANOVA analysis of microbial communities based on baseline characteristics (sex, age, BMI) revealed no significant impact on bacterial or fungal communities (PERMANOVA, FDR-P > 0.05; **Supplementary Table 1**).

**Table 1:** **Overview of study cohort characteristics**.

Baseline characteristic	NC		C3M		C6M		Pvalue
	Number	Percent or SD	Number	Percent or SD	Number	Percent or SD	
Number of participants	51	100%	27	100%	41	100%	
Age, mean years	44.9	11.4	41.7	7.59	45.7	11	0.12
sex							0.03
Famale	28	52.80%	15	55.60%	33	80.50%	
Male	25	47.20%	12	44.40%	8	19.50%	
BMI, mean	23.2	2.7	24.2	3.4	22.9	3	0.18
Smoker							0.35
Yes	11	20.80%	4	14.80%	5	12.20%	
No	42	79.20%	23	85.20%	36	87.80%	
Alcohol drinker							0.001
Yes	17	32.10%	18	66.70%	28	68.30%	
No	36	67.90%	9	33.30%	13	31.70%	

Table describing the study cohort characteristics of the three healthy cohorts: NC, C3M and C6M.

Values are represented in number and percentage or mean and standard deviation. Differences between the groups were tested using two-tailed ANOVA test (for continuous variables) and Chi-square test or Fisher’s exact test (for non-continuous variables).

### 3.2 Long-Term Effects of Mild COVID-19 on Gut Bacterial Communities

**Figure 2:**
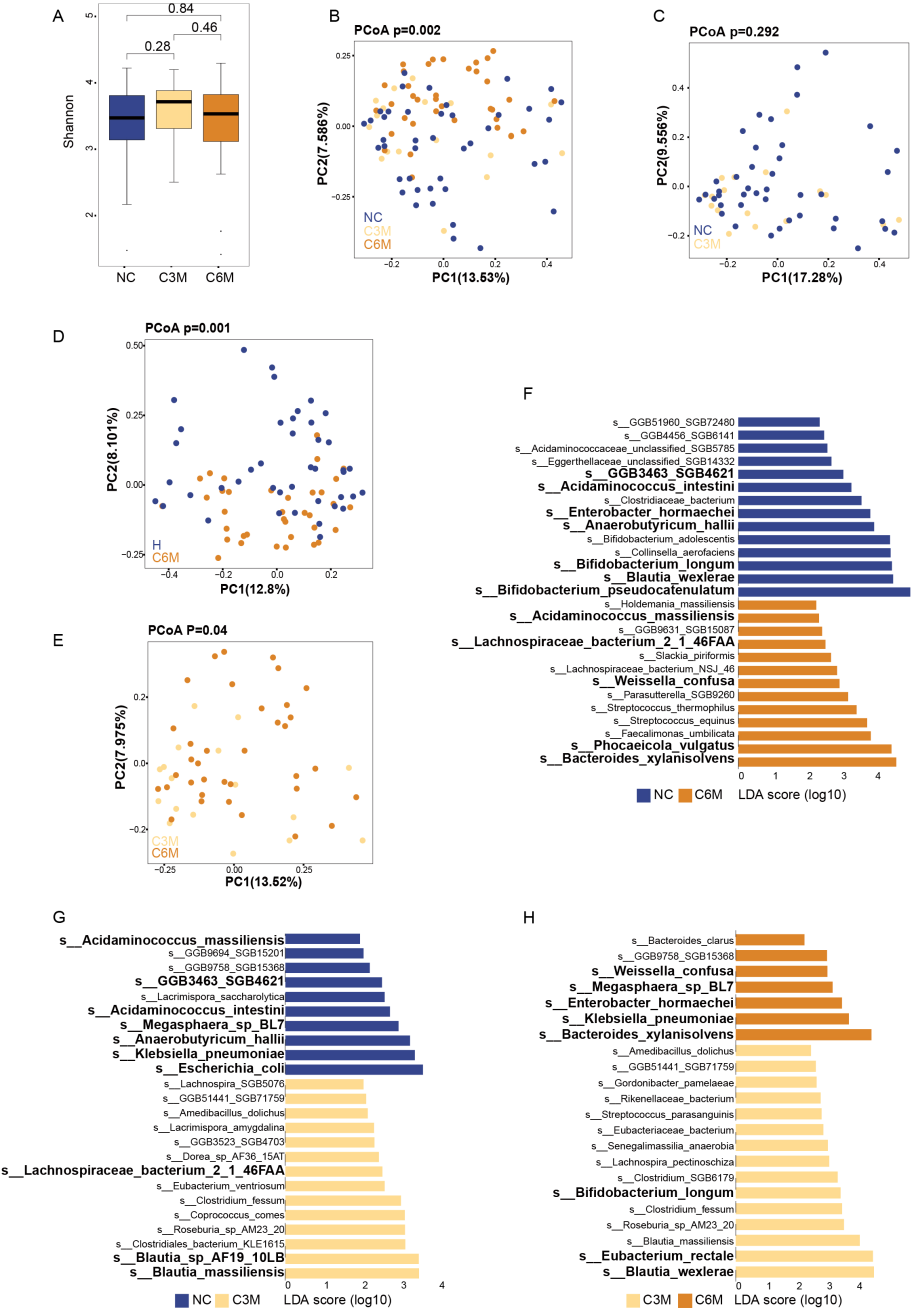
Long-term effects of mild COVID-19 on gut bacterial communities. **(A)** Shannon diversity index comparison between the three healthy cohorts (NC, C3M, C6M). **(B)** Principal coordinates analysis (PCoA) showing beta diversity differences between the three healthy cohorts: NC (n = 51), C3M (n = 27), and C6M (n = 41) subjects. PERMANOVA, FDR-p=0.002. **(C)** Principal coordinates analysis (PCoA) showing beta diversity differences between two healthy cohorts: NC (n = 51) and C3M (n = 27) subjects. PERMANOVA, FDR-p=0.292. **(D)** Principal coordinates analysis (PCoA) showing beta diversity differences between two healthy cohorts: NC (n = 51) and C6M (n = 41) subjects. PERMANOVA, FDR-p=0.001. **(E)** Principal coordinates analysis (PCoA) showing beta diversity differences between two healthy cohorts: C3M (n = 27), and C6M (n = 41) subjects. PERMANOVA, FDR-p=0.04. **(F)** LDA effect size (LDA) plot of bacterial species abundance differences between NC and C3M, analyzed using LEfSe and FDR correction (FDR-P < 0.05). The upper section indicates species enriched in H, while the lower section shows species enriched in C3M. **(G)** LDA effect size (LDA) plot of bacterial species abundance differences between NC and C6M, analyzed using LEfSe and FDR correction (FDR-P < 0.05). The upper section indicates species enriched in NC, while the lower section shows species enriched in C6M. **(H)** LDA effect size (LDA) plot of bacterial species abundance differences between C6M and C3M, analyzed using LEfSe and FDR correction (FDR-P < 0.05). The upper section indicates species enriched in C6M, while the lower section shows species enriched in C3M.

The composition of gut bacterial communities was assessed across the NC, C3M, and C6M groups. No significant differences in alpha diversity were observed among the three groups. Both the Shannon diversity index and species richness showed similar diversity levels across the groups (**Figure 2A**, NC vs. C3M, P = 0.28; C3M vs. C6M, P = 0.46; NC vs. C6M, FDR-P = 0.84). However, a slight trend of increased alpha diversity was observed in the C3M group, followed by a return to NC group levels in the C6M group. Overall, distinct clustering of participants from the C6M, NC, and C3M groups was observed (**Figure 2B**, PERMANOVA FDR-P = 0.002). Importantly, the C3M group clustered separately from the C6M group and was closer to the NC group, as confirmed by pairwise comparisons. No significant beta diversity differences were observed between the NC and C3M groups (**Figure 2C**, PERMANOVA FDR-P = 0.292), but significant differences were found between the NC and C6M groups (**Figure 2D**, PERMANOVA FDR-P = 0.001) and between the C3M and C6M groups (**Figure 2E**, PERMANOVA FDR-P = 0.04). Species-level analysis revealed differences in microbial composition among the groups. In the NC versus C3M comparison, *Escherichia coli* and *Klebsiella pneumoniae* were more abundant in the NC group, while *Blautia massiliensis* and *Blautia* sp. *AF19_10LB* was enriched in the C3M group (**Figure 2F**). In the NC versus C6M comparison, *Blautia wexlerae* and *Bifidobacterium pseudocatenulatum* were more abundant in the NC group, whereas *Bacteroides xylanisolvens* and *Phocaeicola vulgatus* were enriched in the C6M group (**Figure 2G**). In the C3M versus C6M comparison, *Eubacterium rectale* and *Blautia wexlerae* were more abundant in the C3M group, while *Klebsiella pneumoniae* and *Bacteroides xylanisolvens* were enriched in the C6M group (**Figure 2H**). Dynamic changes in bacterial species were also observed. For instance, *Lachnospiraceae bacterium 2_1_46FAA* was absent in the NC group but enriched in the C3M and C6M groups. In contrast, *Anaerobutyricum hallii*, *Acidaminococcus intestini*, and *GGB3463_SGB4621* were enriched in the NC group but absent in the C3M and C6M groups. *Enterobacter hormaechei*, *Acidaminococcus massiliensis*, and *Megasphaera* sp. *BL7* was absent in the C3M group but enriched in the NC and C6M groups. *Weissella confusa* and *Bacteroides xylanisolvens* were enriched in the C6M group but absent in the NC and C3M groups. Conversely, *Bifidobacterium longum* and *Blautia wexlerae* were enriched in the NC and C3M groups but absent in the C6M group. A multifactor PERMANOVA model revealed that microbial beta diversity was influenced by gender and smoker (**Supplementary Table 1**), though these factors did not explain the differences between the NC and C6M groups. When restricting the analysis to the male participants, significant differences between the NC and C6M groups were observed (PERMANOVA FDR-P = 0.019), while no significant smoker differences were detected (PERMANOVA FDR-P = 0.803).

### 3.3 Long-term effects of mild COVID-19 on gut fungal communities

**Figure 3:**
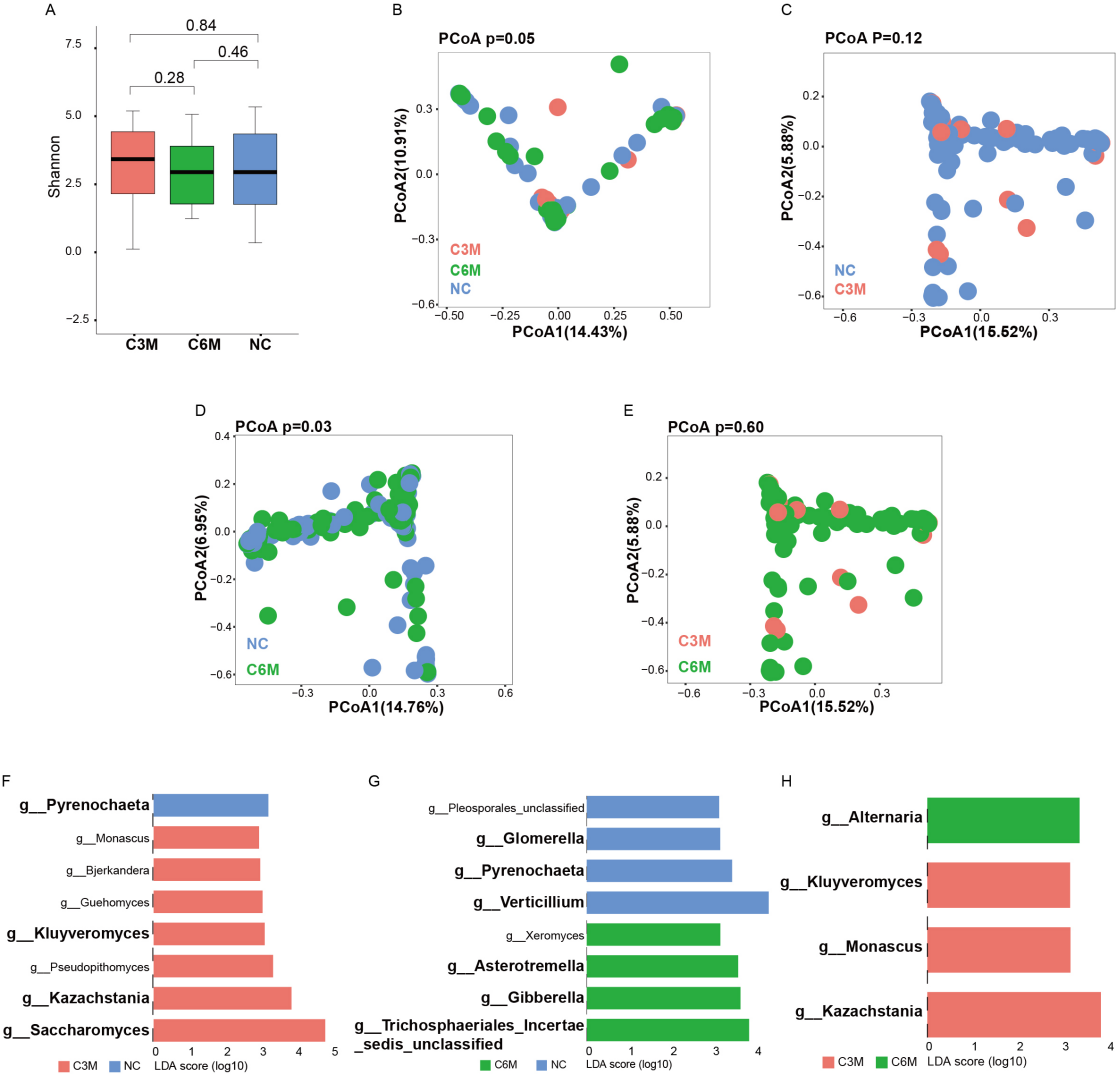
Long-term effects of mild COVID-19 on gut fungal communities. **(A)** Shannon diversity index comparison between the three healthy cohorts (NC, C3M, C6M). **(B)** Principal coordinates analysis (PCoA) showing beta diversity differences between the three healthy cohorts: NC (n = 51), C3M (n = 27), and C6M (n = 41) subjects. PERMANOVA, FDR-p=0.05. **(C)** Principal coordinates analysis (PCoA) showing beta diversity differences between two healthy cohorts: NC (n = 51) and C3M (n = 27) subjects. PERMANOVA, FDR-p=0.12. **(D)** Principal coordinates analysis (PCoA) showing beta diversity differences between two healthy cohorts: NC (n = 51) and C6M (n = 41) subjects. PERMANOVA, FDR-p=0.03. **(E)** Principal coordinates analysis (PCoA) showing beta diversity differences between two healthy cohorts: C3M (n = 27), and C6M (n = 41) subjects. PERMANOVA, FDR-p=0.6. **(F)** LDA effect size (LDA) plot of fungal genera abundance differences between NC and C3M, analyzed using LEfSe and FDR correction (FDR-P < 0.05). The upper section indicates species enriched in H, while the lower section shows species enriched in C3M. **(G)** LDA effect size (LDA) plot of fungal genera abundance differences between NC and C6M, analyzed using LEfSe and FDR correction (FDR-P < 0.05). The upper section indicates species enriched in NC, while the lower section shows species enriched in C6M. **(H)** LDA effect size (LDA) plot of fungal genera abundance differences between C6M and C3M, analyzed using LEfSe and FDR correction (FDR-P < 0.05). The upper section indicates species enriched in C6M, while the lower section shows species enriched in C3M.

The gut fungal community composition was similarly analyzed across the NC, C3M, and C6M groups. Consistent with the bacterial findings, no significant alpha diversity differences were observed among the three groups. The Shannon diversity index and observed species richness indicated similar diversity levels (**Figure 3A**, NC vs. C3M, FDR-P = 0.84; NC vs. C6M, FDR-P = 0.46; C3M vs. C6M, FDR-P = 0.28). A slightly elevated alpha diversity trend was noted in the C3M group, followed by a return to NC group levels in the C6M group. In beta diversity analysis, PCoA results showed distinct clustering of the NC, C3M, and C6M groups. Notably, the differentiation in fungal community composition was less pronounced than in the bacterial communities (**Figure 3B**, PERMANOVA FDR-P = 0.05). Significant differences were observed between the NC and C6M groups (**Figure 3D**, PERMANOVA FDR-P = 0.03). but no significant differences were observed between the NC and C3M groups (**Figure 3C**, PERMANOVA FDR-P = 0.12) or between the C3M and C6M groups (**Figure 3E**, PERMANOVA FDR-P = 0.60). At the genus level, differences in fungal taxa were observed between the groups. In the NC versus C3M comparison, *Pyrenochaeta* was most enriched in the NC group, while *Saccharomyces* and *Kazachstania* were more abundant in the C3M group (**Figure 3F**). In the NC versus C6M comparison, *Verticillium* and *Pyrenochaeta* were enriched in the NC group, whereas *Gibberella* and *Trichosphaeriales Incertae sedis unclassified* were more abundant in the C6M group (**Figure 3G**). In the C3M versus C6M comparison, *Kazachstania* and *Monascus* were enriched in the C3M group, while *Alternaria* was more abundant in the C6M group (**Figure 3H**). Dynamic changes in fungal taxa were also observed. *Pyrenochaeta* was enriched in the NC group but absent in the C3M and C6M groups. *Kazachstania* and *Kluyveromyces* were enriched in the C3M group but absent in the NC and C6M groups.

### 3.4 Correlation of bacterial and fungal features with blood parameters

**Figure 4:**
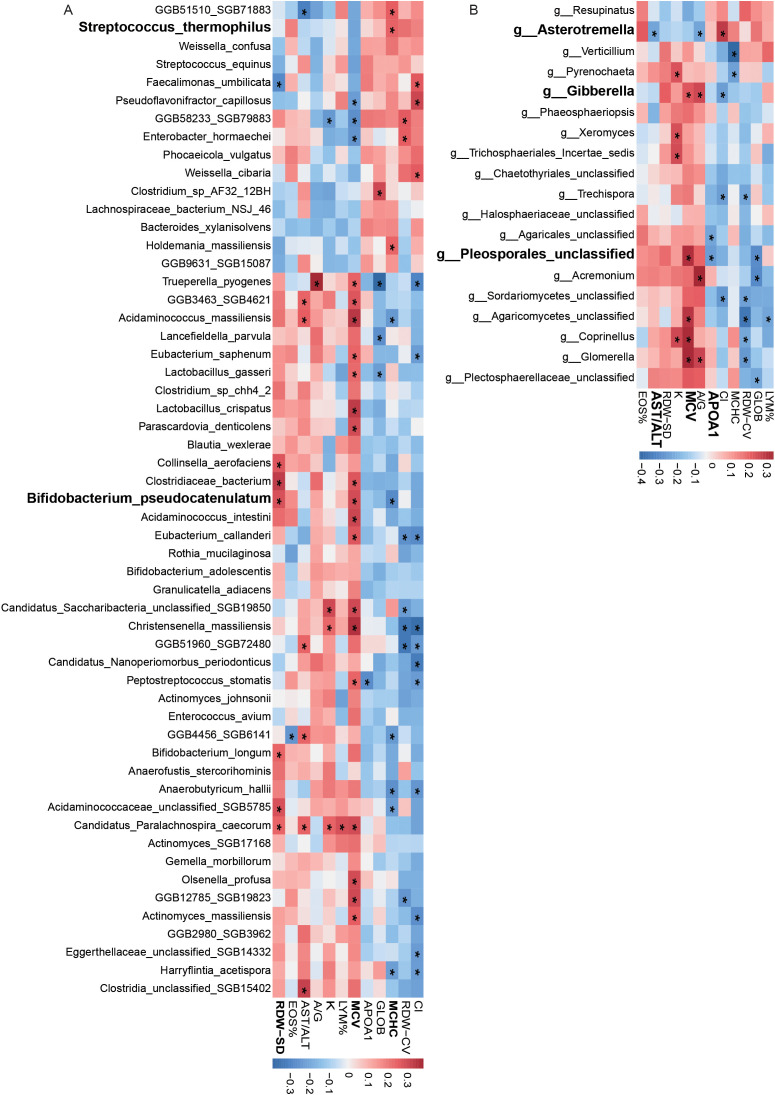
Correlation of bacterial and fungal features with blood parameters. **(A)** Correlations between bacterial species abundance and blood biochemical measurements. **(B)** Correlations between fungal species abundance and blood biochemical measurements.

Bacterial features showed significant correlations with blood parameters. For example, *Streptococcus thermophilus* was positively correlated with mean corpuscular hemoglobin concentration (MCHC), and *Bifidobacterium pseudocatenulatum* was positively correlated with red cell distribution width (RDW-SD) and mean corpuscular volume (MCV) (**Figure 4A**). Fungal features also demonstrated significant associations. For example, *Asterotremella* was negatively correlated with the ratio of aspartate aminotransferase to alanine aminotransferase (AST/ALT) levels, while *Gibberella* was positively correlated with MCV and the albumin/globulin (A/G) ratio (**Figure 4B**). In addition, certain fungi, such as *Pleosporales_unclassified*, were significantly negatively correlated with apolipoprotein A1 (APOA1).

### 3.5 Co-occurrence network analysis of gut bacteria and fungi

**Figure 5:**
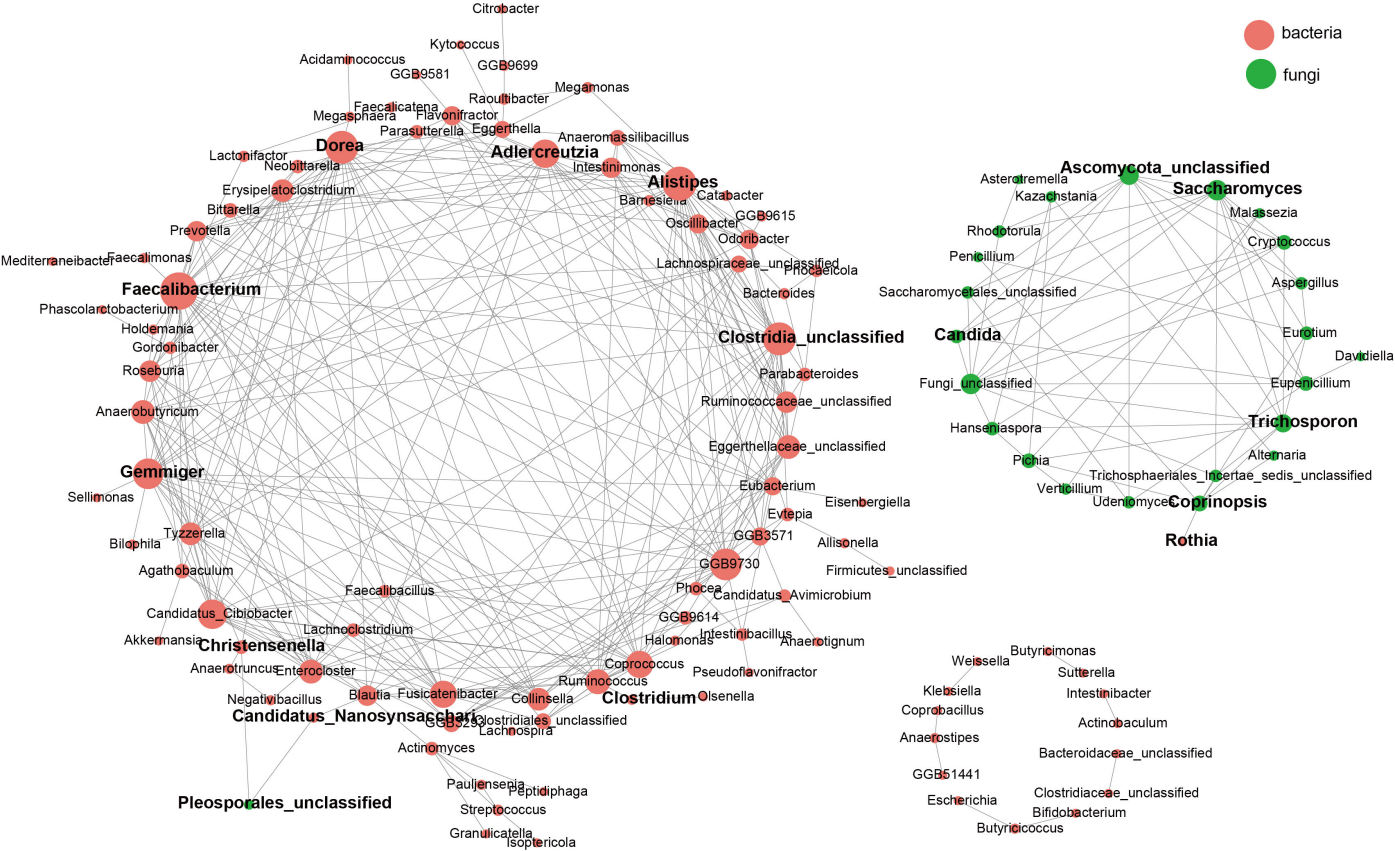
Correlations between the gut mycobiome and bacterial microbiome. Correlations among gut fungal and bacterial species. Correlations among taxa were calculated through the SpeciEasi correlation test. The correlation coefficient was calculated, and statistical significance was pairwise determined for all comparisons. Only statistically significant correlations with jcorrelation coefficient > 0.2 were plotted. The correlation network was visualized via Cytoscape. The size of the node, corresponding to microbial species, was proportional to the number of significant interspecies correlations.

Co-occurrence network analysis in recovery groups revealed complex interactions between bacteria (red nodes) and fungi (green nodes) (**Figure 5**). Central bacterial taxa included *Bacteroides*, *Prevotella*, and *Alistipes*, with opportunistic pathogens such as*Clostridium difficile* also identified. Key fungal taxa, including *Candida* and *Saccharomyces*, exhibited strong connectivity, with *Candida* forming intricate associations with multiple bacterial species. Positive correlations between specific bacteria and fungi, such as *Coprinopsis* and *Rothia*, highlighted significant symbiotic relationships.

### 3.6 ROC curve analysis

**Figure 6:**
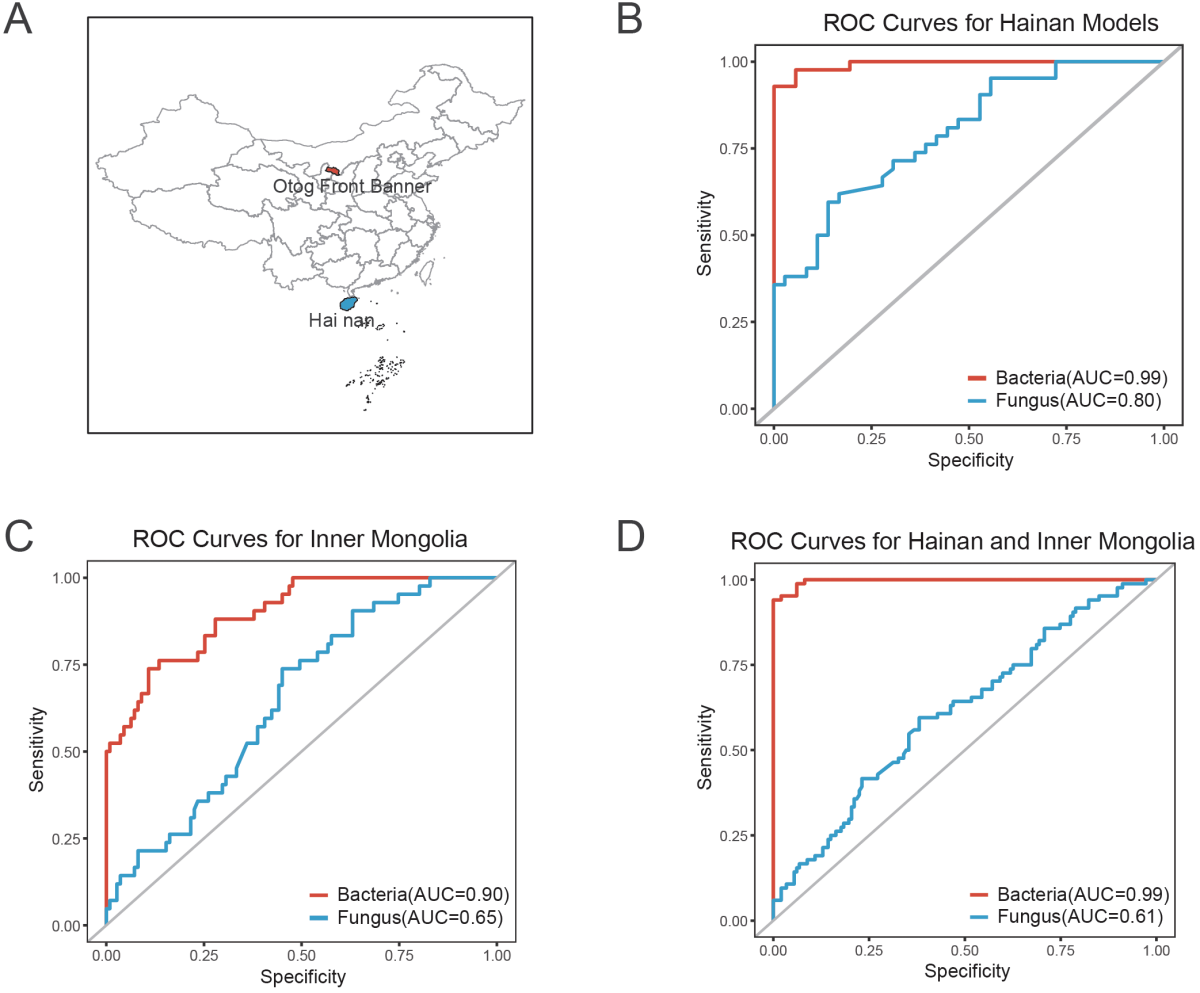
ROC curve analysis and sampling sites for predicting COVID-19 recovery status based on bacterial and fungal features. **(A)** Map showing the sampling sites for the Hainan and Inner Mongolia cohorts. The geographic locations of both regions are indicated to provide context for the study cohorts. **(B)** ROC curve for the bacterial and fungal classification models in the Hainan cohort. The bacterial model achieved an AUC of 0.99, indicating perfect predictive ability, while the fungal model achieved an AUC of 0.80, demonstrating high accuracy. **(C)** ROC curve for the bacterial and fungal models in the Inner Mongolia cohort. The bacterial model achieved an AUC of 0.90, showing near-perfect predictive performance, while the fungal model achieved an AUC of 0.65, maintaining strong discriminatory ability. **(D)** Combined ROC curve for both the Hainan and Inner Mongolia cohorts, illustrating cross-regional validation. The bacterial model demonstrated exceptional robustness with an AUC of 0.99, while the fungal model achieved an AUC of 0.61, indicating high performance across both regions.

To evaluate the discriminatory power of bacterial and fungal features in predicting COVID-19 recovery status, classification models were constructed using 10 bacterial taxa (e.g., *Bifidobacterium pseudocatenulatum*, *Bifidobacterium longum*, *Phocaeicola vulgatus*) and 8 fungal taxa (e.g., *Asterotremella*, *Gibberella*, *Glomerella*). ROC curve analysis was performed separately for the Hainan and Inner Mongolia cohorts (**Figure 6A**, **Supplementary Table 2**). In the Hainan cohort, the bacterial model achieved an AUC of 0.99, demonstrating perfect predictive ability, while the fungal model achieved an AUC of 0.80 (**Figure 6B**). In the Inner Mongolia cohort, the bacterial model achieved an AUC of 0.90, slightly lower but still near-perfect, while the fungal model achieved an AUC of 0.65 (**Figure 6C**). The bacterial model consistently outperformed when combining both cohorts for cross-regional validation, with an AUC of 0.99, demonstrating exceptional accuracy and robustness. The fungal model achieved an AUC of 0.61, bacterial model maintaining high predictive performance (**Figure 6D**). 

## 4 Discussion

This study is the first to explore changes in gut bacterial and fungal communities in individuals recovering from mild COVID-19. In a cohort from Hainan Province, we found significant alterations in the gut microbiota of individuals 3 months (C3M) and 6 months (C6M) after recovering from mild COVID-19, compared to those who had never been infected (NC group). These changes persisted even 6 months post-recovery. Additionally, the predictive performance of characteristic bacterial features was validated in the C6M group using a cohort from Inner Mongolia, confirming the generalizability of COVID-19’s long-term impact on the gut microbiota. These findings suggest that COVID-19 may contribute to gastrointestinal symptoms and metabolic disorders by altering the composition of gut bacteria and fungi. Notably, changes in characteristic microbial taxa were not significantly associated with factors such as age, gender, or BMI, indicating a degree of universality.

Our findings advance the understanding of post-COVID microbiome recovery through three principal innovations that address critical limitations in existing literature. First, whereas prior investigations predominantly characterized severe cases (Chen et al., 2022) and bacterial monocultures (Upadhyay et al., 2023), our integration of fungal dynamics with longitudinal sampling reveals stage-specific mycobiome regulation (**Figures 3F-G**). The *Kluyveromyces*-to-*Asterotremella* transition demonstrates fungal succession temporally coordinated with metabolic recovery (AST/ALT ratio reduction: β=-0.42, 95%CI -0.68 to -0.16; P=0.008), a dimension absent in bacterial-centric frameworks (Su et al., 2024). Second, contrasting with presumed geography-driven microbiota instability (Kopera et al., 2024), we demonstrate bacterial signature robustness across 15° latitude through independent validation: the 10-taxa bacterial model (*Bifidobacterium pseudocatenulatum*, *Phocaeicola vulgatus*, etc.) maintained near-perfect performance in both tropical Hainan (AUC=0.99, 95%CI 0.97-1.00; **Figure 6B**) and temperate Inner Mongolia (AUC=0.90, 95%CI 0.84-0.96; **Figure 6C**), outperforming all existing single-region models (Yeoh et al., 2021; Su et al., 2024). Third, while fungal communities showed geographical variability (Hainan AUC=0.80 vs. Inner Mongolia AUC=0.65; **Figures 6B-C**), their combined predictive capacity (AUC=0.61, 95%CI 0.54-0.68; **Figure 6D**) nevertheless exceeded chance-level discrimination (DeLong test P=0.007), suggesting complementary prognostic value when integrated with bacterial features. This dual-domain approach achieved unprecedented cross-regional accuracy (combined bacterial AUC=0.99; **Figure 6D**), establishing a new paradigm for post-viral microbiome monitoring.

Consistent with previous studies, our research confirmed that mild COVID-19 infection induces significant changes in the gut bacterial community, which persist up to 6 months post-recovery (Zuo et al., 2020; Kim et al., 2021; Lu et al., 2021; Neurath et al., 2021; Xu et al., 2021; Yeoh et al., 2021; Zuo et al., 2021; Bacorn et al., 2022; Chen et al., 2022; Romani et al., 2022; Suskun et al., 2022; Xiang and Liu, 2022; Upadhyay et al., 2023). While alpha diversity metrics such as the Shannon index indicate partial recovery, overall diversity did not return to pre-infection levels. Several probiotics were enriched in the C3M and C6M groups, suggesting a potential role in gut barrier function, immune regulation, and metabolic recovery. For instance, in the C3M group, probiotics such as *Blautia massiliensis*, *Eubacterium ventriosum*, and *Coprococcus comes* were significantly enriched. *Blautia massiliensis* has been associated with metabolic improvement and weight recovery in adolescents (Hou et al., 2023; Mostafa et al., 2024). Our KEGG functional analysis further supports the metabolic role of *Blautia massiliensis*, showing its involvement in tryptophan metabolism. This suggests that *Blautia massiliensis* may contribute to metabolic recovery, potentially by modulating amino acid metabolism, which is essential for maintaining overall metabolic balance. Similarly, *Eubacterium ventriosum* and *Coprococcus comes* produce short-chain fatty acids (SCFAs) like butyrate through the fermentation of dietary fiber, contributing to gut barrier integrity, immune modulation, and reducing the risk of colon cancer, obesity, and type 2 diabetes (Mohr et al., 2022; Jardon et al., 2024; Lin et al., 2024; Nayman et al., 2024; Nechalová et al., 2024; Schwenger et al., 2024).

In the C6M group, probiotics such as *Weissella confusa*, *Phocaeicola vulgatus*, and *Streptococcus thermophilus* were prominently enriched. These taxa are associated with anti-inflammatory and antioxidant effects, metabolic liver disease improvement, and restored gut microbial balance (Vitetta et al., 2019; Wang et al., 2022; Noh et al., 2023; Yang et al., 2023; Jin et al., 2024). Our KEGG functional analysis further supports the anti-inflammatory role of *Weissella confusa*, revealing its involvement in the Bifidobacterium shunt pathway. This pathway is known to contribute to the reduction of inflammation, providing additional evidence for the potential anti-inflammatory effects of *Weissella confusa* in the post-COVID recovery phase. Despite the gradual recovery of probiotics in the C6M group, certain opportunistic pathogens, such as *Streptococcus equinus* and *Slackia piriformis*, remained enriched, indicating potential long-term health risks. These pathogens have been associated with conditions like colonic tumors and anemia, warranting further investigation (Dekker and Lau, 2016; Wang et al., 2022; Liang et al., 2024). Dynamic bacterial changes during recovery reflected the microbiome reconstruction process. For example, *Lachnospiraceae bacterium 2_1_46FAA* was enriched in both the C3M and C6M groups, suggesting a potential role in the recovery phase. However, its abnormal enrichment in studies on impaired glucose tolerance indicates a possible involvement in metabolic dysfunction, highlighting the need for further investigation (Zhang et al., 2024). Conversely, *Anaerobutyricum hallii* was enriched in the NC group but significantly depleted in the C3M and C6M groups, consistent with its reduced presence in patients with persistent metabolic disorders during long COVID (Zhang et al., 2023). This dynamic shift in the gut microbiome suggests that the reconstruction of the microbial community over time is influenced by factors such as metabolic dysregulation and immune modulation during recovery. Probiotic supplementation targeting specific taxa, like *Anaerobutyricum hallii*, may help restore microbial balance and alleviate symptoms of long COVID. These findings underscore the importance of continuous monitoring and targeted interventions in managing the long-term effects of mild COVID-19 on gut health.

This study is the first to examine the long-term effects of COVID-19 recovery on gut fungal communities, shedding light on the dynamic shifts in fungal taxa and their potential implications for immune modulation and gut recovery. Fungal communities, particularly their interaction with the immune system, have been increasingly recognized for their role in regulating inflammation and promoting gut barrier function. Compared to the NC group, the probiotic fungus *Kluyveromyces* spp. was enriched in the C3M group, suggesting potential benefits for alleviating both gastrointestinal and systemic symptoms (Navarro-López et al., 2022; Viebahn et al., 2024). For example, *Kluyveromyces marxianus*, in combination with *Lactobacillus rhamnosus*, has shown promise in alleviating post-COVID gastrointestinal symptoms, indicating its potential therapeutic relevance in the post-COVID context (Navarro-López et al., 2022). By the C6M stage, however, the levels of *Kluyveromyces* spp. decreased, and the anti-inflammatory fungus *Asterotremella* spp. became more prominent, signifying a gradual recovery of the fungal microbiome (Guo et al., 2024). This shift from a probiotic to an anti-inflammatory fungal profile may support a balanced immune response and aid in recovery from COVID-19-associated inflammation. However, concerns arise with the persistent enrichment of opportunistic pathogens like *Alternaria* in the C6M group. This genus has been linked to symptom exacerbation following gastroesophageal reflux disease treatment, highlighting the importance of continuous monitoring for pathogenic fungal overgrowth, which may interfere with gut recovery (Shi et al., 2023). Additionally, dynamic changes in fungal taxa, such as the consistent depletion of *Pyrenochaeta* spp. and the transient enrichment of *Kazachstania* spp., further emphasize the incomplete restoration of the gut microbiota, particularly in the early stages of recovery. *Kazachstania* spp. in the C3M group, absent at later stages, might contribute to immune modulation and inflammation regulation, suggesting its potential role in the recovery process (Wang et al., 2022). These findings underscore the complex interplay between fungal dynamics and immune modulation during COVID-19 recovery. Future studies should integrate metabolomics and immunomics approaches to better elucidate the specific mechanisms by which fungal communities influence gut health and immune responses. Understanding these dynamics could offer new insights into post-viral gut recovery strategies and help develop targeted interventions for long-term COVID-19 sequelae.

We further examined the associations between gut microbiota and clinical indicators, highlighting the potential biological functions of specific taxa during COVID-19 recovery. For example, *Streptococcus thermophilus*, which produces lactic acid metabolites, may improve the gut environment, promote iron absorption, and support red blood cell function, as evidenced by its positive correlation with mean corpuscular hemoglobin concentration (MCHC), thereby alleviating anemia commonly observed during recovery (Muñoz Alférez et al., 2019; Taneri et al., 2020). The abundance of the fungus *Asterotremella* showed a negative correlation with the AST/ALT ratio, suggesting its potential role in liver function recovery through the modulation of the gut-liver axis. Furthermore, previous studies have indicated that high abundance of *Asterotremella* is associated with a good response to anti-inflammatory treatment in Crohn’s disease patients, providing strong support for its potential application in inflammation regulation during COVID-19 recovery (Guo et al., 2024). On the other hand, the abundance of *Pleosporales_unclassified* showed a positive correlation with mean corpuscular volume (MCV) and a negative correlation with apolipoprotein A1 (APOA1), suggesting its significant role in lipid metabolism regulation and inflammatory responses during COVID-19 recovery. Therefore, further research is urgently needed to explore the specific mechanisms of *Pleosporales_unclassified* in the gut-systemic metabolism axis. Co-occurrence network analysis revealed both synergistic and antagonistic relationships among gut microbes. For instance, *Rothia* and *Coprinopsis* spp. exhibited significant synergy, potentially providing a “dual defensive barrier” against opportunistic pathogens such as *Moraxella catarrhalis* and *Candida* (Heleno et al., 2014; Stubbendieck et al., 2023). This synergy may protect against the development of long COVID symptoms. Future interventions based on probiotics or prebiotics targeting these relationships could help regulate the microbiome, reduce chronic inflammation, and support COVID-19 recovery. Finally, random forest and ROC curve analyses evaluated the predictive power of bacterial (*Bifidobacterium pseudocatenulatum*, *Bifidobacterium longum*, *Phocaeicola vulgatus*, etc.) and fungal (*Asterotremella*, *Gibberella*, *Glomerella*, etc.) features in identifying COVID-19 recovery status. The bacterial model outperformed the fungal model, with AUCs of 0.99 and 0.80, respectively. These results were validated in both the Hainan and Inner Mongolia cohorts, demonstrating strong cross-regional applicability. This validation framework provides a new perspective for microbiome biomarker studies. The machine learning models developed in this study offer promising clinical applications, particularly in predicting post-COVID-19 recovery trajectories based on gut microbiota profiles. By analyzing the microbial features identified through our random forest model, healthcare providers could assess which patients are at higher risk of prolonged microbiota imbalances or related health issues, and provide personalized interventions. For instance, the model’s ability to predict recovery status could guide clinicians in recommending specific probiotics or dietary changes to support gut restoration and metabolic recovery. Future research integrating metabolomics and functional genomics could provide further insights into the mechanisms of key taxa, offering precise targets for microbiome-based personalized interventions.

Although this study highlights significant changes in the gut microbiota following COVID-19 recovery and their associations with clinical parameters, its cross-sectional design limits the ability to draw causal conclusions. Longitudinal studies are needed to monitor microbiome changes over time following recovery. Additionally, this study primarily focuses on healthy, excluding those with long COVID, which restricts the generalizability of the findings. Further research should expand the sample scope and explore the efficacy of probiotics or dietary interventions in restoring microbial balance and alleviating long COVID symptoms (Collaborators GUBoD, 2024).

## Data Availability

The datasets presented in this study can be found in online repositories. The names of the repository/repositories and accession number(s) can be found below: https://www.ncbi.nlm.nih.gov/, PRJNA1182485.
