# Supplementary material for: Long-term alterations in gut microbiota following mild COVID-19 recovery: bacterial and fungal community shifts
Source: Front Cell Infect Microbiol. 2025 May 26;15:1565887. doi: 10.3389/fcimb.2025.1565887 (PMC12146308; doi:10.3389/fcimb.2025.1565887)
Supplement: Supplementary file 1 [file Table1.docx]

| Bacteria(Spacies) | Bacteroides_xylanisolvens |
| --- | --- |
| Bifidobacterium_pseudocatenulatum | Verticillium |
| Bifidobacterium_longum | Trichosphaeriales_Incertae_sedis_unclassified |
| Phocaeicola_vulgatus | Gibberella |
| Anaerobutyricum_hallii | Asterotremella |
| Blautia_wexlerae | Xeromyces |
| GGB4456_SGB6141 | Glomerella |
| Holdemania_massiliensis | Pyrenochaeta |
| Collinsella_aerofaciens | Pleosporales_unclassified |
| Parasutterella_SGB9260 |  |
| Bacteroides_xylanisolvens |  |

**Supplementary table 2: Key Microbial Features Identified by Random Forest and ROC Curve Analysis** This table lists the specific bacterial and fungal species used to construct the ROC curve model for predicting COVID-19 recovery status. These species were identified as key microbial features that can predict recovery status following mild COVID-19 infection.
